# Mobility of Indaziflam Influenced by Soil Properties in a Semi-Arid Area

**DOI:** 10.1371/journal.pone.0126100

**Published:** 2015-05-07

**Authors:** Amir M. González-Delgado, Jamshid Ashigh, Manoj K. Shukla, Russ Perkins

**Affiliations:** 1 Plant and Environmental Sciences Department, New Mexico State University, P.O. Box 30003 MSC 3Q Skeen Hall Room N 127, Las Cruces, New Mexico, 88003–8003, United States of America; 2 Department of Entomology, Plant Pathology and Weed Science, New Mexico State University, P.O. Box 30003 MSC 3AE Skeen Hall Room N 140, Las Cruces, New Mexico, 88003–8003, United States of America; 3 Bayer CropScience LP, Field Development-Southern, P.O. Box 146 916 9^th^ Street, Idalou, Texas, 79329, United States of America; Banaras Hindu University, INDIA

## Abstract

Indaziflam, a broad-spectrum, pre-emergence herbicide was the focus of a field investigation conducted after the identification of sporadic injury symptoms on the pecan trees a few months after the application. The study was conducted in two pecan orchards located in southern New Mexico, USA, and southeastern Arizona, USA. The objectives of this study were to evaluate the occurrence and distribution of indaziflam in the soil profile of areas where pecan trees were injured (impacted) and areas where no injury symptoms were observed (unimpacted), and to determine the relationship between indaziflam concentrations and soil properties in those locations. Soil samples were collected, one year after applications, from six depth representing 0–7, 7–15, 15–30, 30–60, 60–90 and 90–120 cm depth to determine the concentration of indaziflam in impacted and unimpacted areas of the two orchards. Soil samples were analyzed to determine texture, bulk density, organic matter content, cation exchange capacity, pH, nitrate, chloride and calcium concentrations. The detection frequency of indaziflam was higher in Arizona than in New Mexico, likely due to the differences between the tillage practices and sand contents of the orchards. No significant correlations were observed between indaziflam and soil properties, however indaziflam was mostly detected in areas where pecan trees were unimpacted probably as result of greater organic matter content and soil porosity. More research is needed to understand the causes of injury to pecan trees by indaziflam application.

## Introduction

Indaziflam (N-[(1R,2S)-2,3-dihydro-2,6-dimethyl-1H-inden-1-yl]-6-[(1R)-1-fluoroethyl]-1,3,5-triazine-2,4-diamine) is an alkylazine herbicide manufactured by Bayer CropScience. This herbicide is a potent inhibitor of cellulose biosynthesis and is used for pre-emergence control of annual grass and broadleaf weeds [1]. Indaziflam is currently registered or being registered for use in perennial crops (e.g., citrus, tree nut, grapes, pome and stone fruit), residential and commercial areas (e.g., turfgrass, landscape ornamentals, Christmas trees, hardscapes), non-residential and non-crop areas (e.g., railroad and rail yards, roadsides, fence rows, industrial sites), and forestry sites. Indaziflam provides long-lasting residual activity at low application rates, due to its long persistence in soil (*t*_*1/2*_ = 150 days) [2], however other studies reported t_1/2_ = 22–176 days after conducting studies with 4 and 2 soils from Europe and United States of America, respectively [3].

Previous studies indicate that indaziflam is a weak acid and anionic at the soil pH values of 5.4 and above [1, 2]. Indaziflam is not volatile and its dissipation in the environment takes place primarily through degradation and leaching. Indaziflam is classified as moderately mobile in the soil, however its breakdown products (indaziflam-carboxylic acid, fluoroethyldiaminotriazine and fluoroethyltriazinanedione) are more mobile [2]. The water solubility of indaziflam is 0.0028 g/L at 20°C and its organic carbon sorption coefficient (K_oc_) is <1,000 mL/g [2, 4].

Batch experiments indicated that indaziflam is low to moderately mobile in the soil and its sorption was positively correlated with the organic carbon content in six Brazilian oxisols and three U.S. mollisols [1]. Jones et al. [5] evaluated the effect of organic matter content on hybrid bermudagrass (*Cynodon dactylon* x *Cynodon transvaalensis*) injury following the application of indaziflam in minirhizotron cultures. Hybrid bermudagrass injuries decreased with increasing organic matter content. This study indicated that the phytotoxicity effects of indaziflam are greater in soils with low organic carbon content. In a greenhouse study, [6] indicated that the foliar injury and reductions in root-length density of hybrid bermudagrass from indaziflam were greatest at rooting depth of 5 cm than 10 to 15 cm. Furthermore, the study revealed more indaziflam injuries in hybrid bermudagrass established in sand with no organic carbon than in silt loam with organic carbon.

Mobility of indaziflam was compared with other soil-applied herbicides and the effect of the amount of rainfall on leaching was determined in columns repacked with sand [7]. Based on the observed injuries of the ryegrass (*Lolium multiflorum*) planted in the columns, indaziflam was reported to leach to 30 cm depth. Jhala et al. [8] reported that the leaching depth of indaziflam is positively correlated with the application rate and the amount of rainfall.

The aforementioned studies were conducted under laboratory conditions that may not be representative of the field conditions that influence the environmental fate and transport of indaziflam. The fate and transport behavior of pesticides under field conditions are variable because of the temporal and spatial soil variability [9, 10, 11]. Indaziflam is considered a persistent herbicide in the environment [1, 9], however, there is limited or no information on the dissipation of indaziflam under field conditions. Bayer CropScience [3] conducted field studies to determine the dissipation of indaziflam at 5 bare grounds and tree turf covered grounds. Studies are required to characterize the fate and transport behavior of indaziflam at different scales and under different conditions to generate information that would support the future registrations of this herbicide.

In 2012, indaziflam (Alion herbicide, Bayer CropScience, Research Triangle Park, North Carolina) was registered for use in pecan orchards in New Mexico, Arizona, and several other States in the United States. Immediately after indaziflam was registered, several growers applied indaziflam for season-long broad spectrum weed control in their orchards. However, approximately 3 to 4 months (August/September 2012) after application a small number of growers, in New Mexico and Arizona, reported sporadic herbicide injury symptoms on the pecan trees that included necrosis of leaves and varying trunk injuries. Since the affected trees and the severity of injury symptoms in pecan orchards were sporadic, it was hypothesized that the injury symptoms on pecan trees are the result of leaching of indaziflam caused by the local variations in soil physical and chemical properties in orchards. Therefore, objectives of this study were to: a) evaluate the occurrence and distribution of indaziflam in the soil profile of impacted and unimpacted areas of two pecan orchards located in southern New Mexico and south eastern Arizona, one year after indaziflam application, and 2) develop relationships between indaziflam concentrations and soil properties in those locations.

## Materials and Methods

### Study sites

Two pecan orchards, one located in southern New Mexico, USA (NM orchard, 32.412877N, -106.853516W), and the second one located in Arizona, USA (AZ orchard, 32.044169N, -109.707928W) were selected for this study. Indaziflam was broadcasted, by orchard managers, on NM and AZ orchards at its recommended field rate of 73.1 g ai ha^-1^ (5 fl oz of product per acre), on May 8, 2012 and May 15, 2012, respectively. Orchards were irrigated no later than 3 days after herbicide applications. NM orchard was flood-irrigated while, AZ orchard was sprinkler-irrigated. Between indaziflam application day and soil sampling day in NM orchard, approximately 102 cm of irrigation water was applied and 11 cm of precipitation was recorded. In AZ orchard, approximately 80 cm of irrigation and 27 cm of precipitation were recorded between the indaziflam application day and the soil sampling day. The AZ orchard was tilled before the herbicide application; however, no tillage operations were performed after the application.

### Collection and analysis of soil samples

No specific permissions were required and orchard managers agreed with the collection of soil samples. No endangered or protected species were involved in this study. Soil samples were collected from 1.5 m^2^ area near injured (impacted areas) and uninjured (unimpacted areas) pecan trees in both orchards using the core method [12]. Soil samples were collected from six depths (0–7, 7–15, 15–30, 30–60, 60–90 and 90–120 cm depth) at each sampling location. A total of 36 soil samples were collected from NM orchard on March 20, 2013. Eighteen (18) soil samples were collected from the impacted areas and the same number of samples was collected from the unimpacted areas. A total of 35 soil samples from same six depths were collected from AZ orchard on April 24, 2013. Soil samples were not collected from the 90–120 cm depth of the impacted area of the AZ orchard because the gravelly material inhibited the penetration of the soil core sampler. Sampling was done nearly one year after the application to quantify the indaziflam residues at various depths in the soil of impacted and unimpacted areas.

All soil samples were air-dried and passed through 2 mm sieve [13]. Soil particle size distribution was determined using hydrometer method [14] and soil bulk density using core method [12]. Soil chemical properties including cation exchange capacity (CEC), pH, nitrate, chloride, calcium and organic matter content were determined at Harris Lab, Columbus, Nebraska. The concentration of indaziflam in different soil depths of the impacted and unimpacted areas of both orchards was determined in Bayer CropScience laboratory (Research Triangle Park, North Carolina).

Indaziflam was extracted from soil by adding acetonitrile:water (80:20v/v) to a soil aliquot and extracting indaziflam from the sample using microwave assisted extraction. A sample aliquot was amended with an isotopic standard of indaziflam and diluted with deionized water prior to analysis. Samples were analyzed by tandem mass spectrometry (LC/MS/MS) with quantification based on the use of internal standards and comparison of peak areas with those of known standards.

### Statistical analysis

Correlation analyses were conducted to determine the relationship between soil properties and indaziflam concentration. One-way ANOVA was performed to determine the differences in soil properties by depth and among impacted and unimpacted areas using SAS 9.2 (SAS Institute Inc., Cary, NC, USA).

## Results and Discussion

### Occurrence and distribution of indaziflam

The analytical data of soil samples collected from NM and AZ orchards showed traces of indaziflam in both pecan orchards nearly one year after the application (**Table 1**). Indaziflam was detected in only one of the three replicates collected at 7–15 cm depth from the unimpacted areas of NM orchard. The presence of indaziflam at 7–15 cm depth and not at the soil surface could be due to low bioavailability of indaziflam for biodegradation, absence of suitable environmental conditions to promote the degradation process, and sorption of indaziflam to the organic matter or clay at that depth [9]. No indaziflam was detected in the rest of the samples collected from unimpacted and impacted areas of NM orchard. Differences in soil properties could explain the occurrence of indaziflam generally in the unimpacted areas. The organic matter content, clay content and pH play an important role on the sorption of herbicides in the soil [15], therefore these soil properties were evaluated to explain the distribution in the unimpacted and impacted areas. No significant difference in soil pH values was detected between the unimpacted and impacted areas of NM orchard, however soil pH values were greater than 7 in both areas. Indaziflam is a weak acid and anionic at the soil pH values of 5.4 and above [1, 2], therefore the sorption of indaziflam in the unimpacted and impacted areas was expected to be limited. The average organic matter (0.65±0.14%) and average clay (11.93±2.56%) contents in the unimpacted areas were higher and significantly different (p<0.05) compared to the average organic matter (0.53±0.12%) and average clay (7.20±3.17%) contents from the impacted areas (**Table 2**). The greater organic matter and clay content could explain the occurrence of indaziflam in the unimpacted area due to sorption, while indaziflam was not detected in the impacted area probably as result of lower sorption potential. Durovic et al. [16] reported that the sorption of pesticides (atrazine, acetochlor, oxyfluorfen, pendimethalin and clomazone) was influenced by the soil clay and organic carbon contents. These herbicides have different modes of action compared to indaziflam and the K_oc_ of atrazine (100–200 mL/g), acetochlor (314 mL/g), and clomazone (554 mL/g) suggest that they are mobile while oxyfluorfen (5,450 mL/g) and pendimethalin (17,200 mL/g) have a low leaching potential [17, 18, 19, 20, 21]. Due to limited detectable amount of indaziflam in NM orchard, the distribution pattern of indaziflam was not determined. This may be explained by the half-life of indaziflam, *t*_*1/2*_ = 150 days [2]; *t*_*1/2*_ = 22–176 days [3] because samples were collected nearly 365 days after the application. Non detection of indaziflam for a majority of samples is good for maintaining the soil and groundwater quality.

**Table 1:** Indaziflam concentration in soil samples collected from NM orchard and AZ orchard.

				Indaziflam (ug/Kg)	
Site	Sample	Depth (cm)	Replicate 1	Replicate 2	Replicate 3
NM orchard	NM-U-A^c^	0–7	ND^a^	ND^a^	ND^a^
	NM-U-B^c^	7–15	ND^a^	ND^a^	2.6
	NM-U-C^c^	15–30	ND^a^	ND^a^	ND^a^
	NM-U-D^c^	30–60	ND^a^	ND^a^	ND^a^
	NM-U-E^c^	60–90	ND^a^	ND^a^	ND^a^
	NM-U-F^c^	90–120	ND^a^	ND^a^	ND^a^
NM orchard	NM-I-A^d^	0–7	ND^a^	ND^a^	ND^a^
	NM-I-B^d^	7–15	ND^a^	ND^a^	ND^a^
	NM-I-C^d^	15–30	ND^a^	ND^a^	ND^a^
	NM-I-D^d^	30–60	ND^a^	ND^a^	ND^a^
	NM-I-E^d^	60–90	ND^a^	ND^a^	ND^a^
	NM-I-F^d^	90–120	ND^a^	ND^a^	ND^a^
AZ orchard	AZ-U-A^c^	0–7	2.1	2.6	4.7
	AZ-U-B^c^	7–15	2.1	3.4	2.6
	AZ-U-C^c^	15–30	ND^a^	1.5	ND^a^
	AZ-U-D^c^	30–60	ND^a^	ND^a^	ND^a^
	AZ-U-E^c^	60–90	ND^a^	ND^a^	ND^a^
	AZ-U-F^c^	90–120	ND^a^	ND^a^	ND^a^
AZ orchard	AZ-I-A^d^	0–7	2	ND^a^	ND^a^
	AZ-I-B^d^	7–15	ND^a^	ND^a^	ND^a^
	AZ-I-C^d^	15–30	ND^a^	ND^a^	ND^a^
	AZ-I-D^d^	30–60	ND^a^	ND^a^	ND^a^
	AZ-I-E^d^	60–90	ND^a^	ND^a^	ND^a^
	AZ-I-F^d^	90–120	NS^b^	NS^b^	NS^b^

^a^ND, Non-Detected.

^b^NS, Not Sampled.

^c^U, Unimpacted.

^d^I, Impacted.

**Table 2:** Average values of soil physical-chemical properties and nutrient content of soil samples collected from NM orchard and AZ orchard.

Site	Sample	Depth (cm)	pH^c^	CEC^a^ ^,^ ^c^ (cmol/Kg)	BD^a^ ^,^ ^c^ (g/cm^3^)	OM^a^ ^,^ ^c^ (%)	Clay^c^ (%)	Silt^c^ (%)	Sand^c^ (%)	Texture^b^	NO_3_^-^ ^a^ ^,^ ^c^ (mg/Kg)	Cl^-^ ^a^ ^,^ ^c^ (mg/Kg)	Ca^2+^ ^a^ (mg/Kg)
NM Orchard	NM-U-A^a^	0–7	8.53 a	20.85 a	1.31 a	0.90 a	16.72 a	16.14 a	67.13 f	SL	5.33 a	54.96 a	3,467.66
	NM-U-B^a^	7–15	8.38 a	19.03 a	1.39 a	0.80 ab	8.50 f	10.21 d	81.28 a	LS	5.50 a	41.43 a	3,223.66
	NM-U-C^a^	15–30	8.56 a	17.21 a	1.38 a	0.53 c	10.50 e	12.14 b	77.35 c	SL	3.66 a	40.90 a	2,958.33
	NM-U-D^a^	30–60	8.53 a	18.63 a	1.39 a	0.58 bc	12.57 c	16.14 a	71.28 e	SL	3.16 a	34.66 a	3,222.33
	NM-U-E^a^	60–90	8.40 a	18.78 a	1.40 a	0.60 bc	10.64 d	10.00 e	79.35 b	SL	3.16 a	37.83 a	3,235.83
	NM-U-F^a^	90–120	8.53 a	17.96 a	1.41 a	0.53 c	12.64 b	11.92 c	75.42 d	SL	2.16 a	43.05 a	3,080.50
NM Orchard	NM-I-A^a^	0–7	8.50 a	17.36 a	1.41 a	0.75 a	8.50 b	14.00 b	77.49 d	SL	3.00 a	33.71 a	2,927.33
	NM-I-B^a^	7–15	8.60 a	15.55 a	1.43 a	0.58 ab	12.50 a	14.00 b	73.49 e	SL	3.00a	21.55 a	2,677.83
	NM-I-C^a^	15–30	8.58 a	14.23 a	1.45 a	0.46 b	4.57 d	5.78 e	89.64 b	S	2.00 a	24.33 a	2,480.33
	NM-I-D^a^	30–60	8.46 a	16.15 a	1.43 a	0.56 ab	8.50 b	11.71 c	79.78 c	LS	3.33 a	27.15 a	2,801.50
	NM-I-E^a^	60–90	8.55 a	12.60 a	1.48 a	0.46 b	6.57 c	25.85 a	67.56 f	SL	2.16 a	18.10 a	2,188
	NM-I-F^a^	90–120	8.53 a	12.41 a	1.50 a	0.38 b	2.57 e	5.85 d	91.56 a	S	2.33 a	22.10 a	2,172.33
AZ Orchard	AZ-U-A^a^	0–7	7.35 c	14.63 c	1.30 a	0.88 a	16.64 d	21.71 b	61.64 c	SL	2.50 c	4.93 b	2,639.33
	AZ-U-B^a^	7–15	7.63 bc	13.93 c	1.24 ab	0.68 b	16.72 c	21.64 c	61.64 c	SL	2.16 c	6.73 ab	2,502.16
	AZ-U-C^a^	15–30	7.70 b	14.20 c	1.19 bc	0.56 b	18.72 b	19.64 d	61.64 c	SL	2.50 c	4.83 b	2,474.16
	AZ-U-D^a^	30–60	7.91 b	17.93 a	1.18 bc	0.61 b	22.64 a	23.64 a	53.71 d	SCL	3.50 bc	5.93 ab	3,081.83
	AZ-U-E^a^	60–90	8.26 a	16.75 ab	1.15 c	0.31 c	12.72 f	17.64 e	69.64 a	SL	4.33 b	8.11 ab	2,898.50
	AZ-U-F^a^	90–120	8.31 a	15.21 bc	1.18 bc	0.33 c	14.64 e	21.71 b	63.64 b	SL	10.83 a	9.73 a	2,546.83
AZ Orchard	AZ-I-A^a^	0–7	8.33 b	18.33 a	1.34 bc	0.75 a	18.57 c	17.92 d	63.49 d	SL	2.33 a	5.51 a	3,421.33
	AZ-I-B^a^	7–15	8.43 ab	18.68 a	1.27 c	0.56 b	18.64 b	17.78 e	63.56 c	SL	1.66 a	4.25 ab	3,502.83
	AZ-I-C^a^	15–30	8.53 a	17.55 ab	1.34 cb	0.56 b	18.57 c	21.78 b	59.60 e	SL	1.83 a	3.71 b	3,298.00
	AZ-I-D^a^	30–60	8.53 a	16.28 ab	1.30 c	0.50 b	24.43 a	23.92 a	51.64 f	SCL	2.00 a	4.13 ab	3,013.50
	AZ-I-E^a^	60–90	8.55 a	14.25 b	1.38 b	0.48 b	14.57 e	17.78 e	67.64 a	SL	1.83 a	4.18 ab	2,607.33
	AZ-I-F^a^	90–120	8.57 a	10.17 c	1.45 a	0.45 b	16.28 d	19.92 c	63.78 b	SL	1.50 a	3.90 b	1,819.25

^a^Abbreviations: U, Unimpacted; I, Impacted; CEC, Cation Exchange Capacity; BD, Bulk Density, OM, Organic Matter Content; NO_3_^-^, Nitrate; Cl^-^, Chloride; Ca^2+^,Calcium.

^b^Abbreviation of soil texture: SL, Sandy Loam; LS, Loamy Sand; S, Sand; SCL, Sandy Clay Loam

^c^Means within the columns with no common letters are significantly different based on the least significant difference (LSD) test, p-value <0.05.

All soil samples collected at 0–7 and 7–15 cm depths and one sample collected at 15–30 cm depth from the unimpacted area in AZ orchard contained detectable concentrations of indaziflam. The analytical data showed that the concentration of indaziflam decreased with depth (**Table 1**). Indaziflam was also detected in one of the samples collected at 0–7 cm depth from the impacted area. The soil organic carbon content, clay content and pH values from the unimpacted and impacted areas of the AZ orchard were compared to evaluate the distribution of indaziflam in both areas. The average soil pH (7.86±0.37) in the unimpacted area was lower and significantly different (p<0.05) compared to the average pH (8.49±0.12) in the impacted area. Sorption of indaziflam decreases with increasing soil pH and similar to NM orchards sorption was expected to be low at both locations. The organic matter content and soil texture were not significantly different in the unimpacted and impacted areas in AZ orchard, however the bulk density in the unimpacted area was significantly lower (1.21 ± 0.06 g/cm^3^) than that of the impacted area (1.34 ± 0.07 g/cm^3^) (**Table 2**). Similar trend was observed between the bulk density of the unimpacted (1.38 ± 0.03 g/cm^3^) and impacted (1.45 ± 0.03 g/cm^3^) areas in NM orchard. The differences in soil porosity could have been one of the factors that influenced the distribution of indaziflam by providing more sorption sites that reduced the dissipation of indaziflam through degradation and leaching. Cox et al. [22] evaluated the transport behavior of two herbicides in clay, silty clay and sandy clay loam soils and reported that the retardation factor and attenuation of the breakthrough curve of both herbicides were greater in the clay soil with the highest volume of small pores.

The results showed that the detection frequency of indaziflam was lower in NM orchard than AZ orchard. The soil properties and management practices were evaluated to explain the detection of indaziflam in both orchards. Both NM and AZ orchards received similar amounts of water during the 2012 growing season (i.e., a total of 113 cm and 107 cm of water, respectively) through irrigation and precipitation. However, unlike NM orchard, soil in AZ orchard was tilled before the application of indaziflam. Previous studies have reported that tillage can negatively impact the connectivity of the soil pores that contributes to the movement of water and solutes through the soil profile [23, 24, 25]. In addition to the different soil management practices utilized in each orchard, soil particle distribution was different as well. Sandy loam was the dominant soil texture in both orchards at most depths. However, average sand content (77.61±7.26%) was higher while the average clay content (9.57±3.72%) was lower in NM orchard than the average sand content (61.81±4.85%) and average clay content (17.77±3.18%) in AZ orchard, respectively (**Table 2**). The soil taxonomy class in NM orchard is mixed, thermic Typic Torripsamments. A mixed clay mineralogy is comprised of vermiculite, smectite, fine grained mica, kaolinite and chloride [26]. The negative charge on these clay minerals at the soil pH would not be favorable for the sorption of indaziflam. The soil taxonomy class in the AZ orchard is classified as a coarse-loamy, mixed, superactive, calcareous, thermic Typic Torrifluvents. A mixed clay mineralogy mostly comprised of fine grained mica with small amount of smectite and kaolinite [27]. The saturated hydraulic conductivity (K_sat_) values of the soil in the NM orchard range between 1.40 x 10^–5^ m s^-1^ to 4.20 x 10^–5^ m s^-1^ and were higher compared with the K_sat_ values (1.41 x 10^–6^ m s^-1^ to 4.02 x 10^–6^ m s^-1^) of the soil in the AZ orchard. Non-detection of indaziflam in this study is due to the soil sampling about a year after the application. However, indaziflam could be present in the soil at concentrations below the detection limit [28]. The combination of undisturbed soil pore connectivity as a result of no-tillage and higher sand content in NM orchard with attendant high soil drainage capacity could have contributed to a faster dissipation of indaziflam compared with the AZ orchard.

Previous studies have reported that the effects of herbicides on microbial populations were less significant in the rhizosphere under field conditions compared to the impacts and degradation of herbicides under laboratory conditions. It is likely that the rhizosphere is a more resilient environment as a result of denser microbial populations and continuous nutrient supply through root exudation [29]. Umrit and Ng Kee Kwong [30] reported that herbicide dissipation in the field was more rapid than dissipation under controlled laboratory conditions after evaluating the persistence of herbicides (diuron, acetochlor, hexazinone and atrazine) in a sugarcane plantation. Rouchaud et al. [31] reported that a faster biodegradation of isoxaben, a herbicide with the similar mode of action of indaziflam, was observed in the laboratory and field soils previously treated with isoxaben than untreated soil. They attributed it to the likely adaptation of soil microbial capacity toward isoxaben soil metabolism. Microbial biomass was negatively correlated with dosage of herbicides, however no detrimental effects on soil microorganisms were observed when the recommended field application rate was used [32, 33]. Similar results were obtained by [34] and [35] for chlorsulfuron and rimsulfuron known to be weak acids as indaziflam. Chlorsulfuron and rimsulfuron belong to the group of sulfonylurea herbicide that inhibit the production of brached-chain amino acid by targeting the enzyme acetolactate synthase or acetohydroxy acid synthase present in plants, algae, fungi, bacteria and archaea [36, 37]. However, soil microorganisms are not expected to be impacted by indaziflam since it was designed to inhibit cellulose biosynthesis. Compared to other herbicides no adverse or limited effects of indaziflam on soil biology are expected due to its mode of action and absence of chlorine in the indaziflam molecule, however studies are required to evaluate the potential toxicity of indaziflam under laboratory and field conditions. Therefore, in this study we did not quantify the effect of herbicide on microbial population.

### Correlation of soil properties and indaziflam concentration

In NM orchard, correlation analysis showed that depth was negatively correlated with organic matter contents (r = -0.52, p-value <0.01) while it was positively related with bulk density (r = 0.34, p-value <0.05) (**Table 3).** The decreasing soil organic matter with depth was probably due to low organic matter input to soil surface and low leaching to deeper layers [38]. The increasing bulk density with depth was on accord with decreasing organic matter. Increases in bulk density with depth are reported due to changes in organic matter content, porosity and compaction [39]. Sakin et al. [40] and Curtis and Post [41] also reported a negative correlation between organic matter content and soil bulk density.

**Table 3:** Correlation values of soil parameters of soil samples collected from NM orchard.

	Depth	pH	OM^a^	BD^a^	NO_3_^-^ ^a^	CEC^a^	Cl^-^ ^a^	Clay	Silt	Sand	
Depth	1	-0.02	-0.52	0.34	-0.36	-0.26	-0.17	-0.32	-0.08	0.22	r^c^
		NS	<0.01	<0.05	<0.05	NS	NS	NS	NS	NS	p-value^b^
pH		1	-0.37	0.50	-0.38	-0.54	-0.44	0.01	0.09	-0.07	r^c^
			<0.05	<0.01	<0.05	<0.001	<0.01	NS	NS	NS	p-value^b^
OM			1	-0.75	0.71	0.77	0.72	0.50	0.13	-0.35	r^c^
				<0.001	<0.001	<0.001	<0.001	<0.01	NS	<0.05	p-value^b^
BD				1	-0.63	-0.93	-0.78	-0.53	-0.05	0.30	r^c^
					<0.001	<0.001	<0.001	<0.001	NS	NS	p-value^b^
NO_3_^-^					1	0.58	0.64	0.31	0.02	-0.17	r^c^
						<0.001	<0.001	NS	NS	NS	p-value^b^
CEC						1	0.83	0.51	0.002	-0.26	r^c^
							<0.001	<0.01	NS	NS	p-value^b^
Cl^-^							1	0.51	-0.04	-0.24	r^c^
								<0.01	NS	NS	p-value^b^
Clay								1	0.35	-0.75	r^c^
									<0.05	<0.001	p-value^b^
Silt									1	-0.88	r^c^
										<0.001	p-value^b^
Sand										1	

^a^Abbreviations: OM, Organic Matter Content; BD, Bulk Density; NO_3_^-^, Nitrate; CEC, Cation Exchange Capacity; Cl^-^, Chloride; NS, non-significant.

^b^p-value indicates a significance at <0.05, 0.01 and 0.001 levels.

^c^r, Correlation Coefficient.

The CEC was positively correlated with clay (r = 0.51, p-value <0.01) and organic matter contents (r = 0.77, p-value <0.001) (**Table 3**). Oorts et al. [42] reported that the CEC of clay minerals and soil organic matter content increased with decreasing particle size or increasing specific surface area. The average calcium concentration in the soil profile was 2,870±641 mg/kg and the average pH for the soil profile was 8.5±0.13 that are indicative of the alkalinity of the arid soil. Soil pH was positively correlated with bulk density (r = 0.50, p-value <0.01), [43] also reported that pH is a useful indicator to predict bulk density as soil depth increased. Similar to the NM orchard, no correlation was observed between depth and silt content, but clay content (r = -0.44, p-value <0.01) and organic matter content (r = -0.75, p-value <0.001) were negatively correlated with depth in the AZ orchard (**Table 4**).

**Table 4:** Correlation values of soil parameters and indaziflam concentration of soil samples collected from AZ orchard.

	Depth	pH	OM^a^	BD^a^	NO_3_^-^ ^a^	CEC^a^	Cl^-^ ^a^	Clay^a^	Silt	Sand	Indaziflam	
Depth	1	0.45	-0.75	-0.01	0.57	-0.37	0.34	-0.44	-0.06	0.31	-0.40	r^c^
		<0.01	<0.001	NS	<0.001	<0.05	<0.05	<0.01	NS	NS	NS	p-value^b^
pH		1	-0.56	0.38	-0.01	0.15	-0.13	0.02	-0.25	0.10	-0.17	r^c^
			<0.001	<0.05	NS	NS	NS	NS	NS	NS	NS	p-value^b^
OM			1	0.29	-0.44	0.13	-0.29	0.29	0.15	-0.26	0.35	r^c^
				NS	<0.01	NS	NS	NS	NS	NS	NS	p-value^b^
BD				1	-0.47	-0.31	-0.48	0.05	-0.13	0.03	0.39	r^c^
					<0.01	NS	<0.05	NS	NS	NS	NS	p-value^b^
NO_3_^-^					1	0.03	0.69	-0.33	0.16	0.15	0.08	r^c^
						NS	<0.001	NS	NS	NS	NS	p-value^b^
CEC						1	0.07	0.30	-0.04	-0.17	-0.19	r^c^
							NS	NS	NS	NS	NS	p-value^b^
Cl^-^							1	-0.36	0.01	0.23	-0.02	r^c^
								<0.05	NS	NS	NS	p-value^b^
Clay								1	0.61	-0.93	-0.55	r^c^
									<0.001	<0.001	NS	p-value^b^
Silt									1	-0.86	0.47	r^c^
										<0.001	NS	p-value^b^
Sand										1	-0.25	r^c^
											NS	p-value^b^
Indaziflam											1	

^a^Abbreviations: OM, Organic Matter Content; BD, Bulk Density; NO_3_^-^, Nitrate; CEC, Cation Exchange Capacity; Cl^-^, Chloride; NS, non-significant.

^b^p-value indicates a significance at <0.05, 0.01 and 0.001 levels.

^c^r, Correlation Coefficient.

Similar to NM orchard, soil pH was negatively correlated with organic matter content (r = -0.56, p-value <0.001) and positively with soil bulk density (r = 0.38, p-value <0.05) in AZ orchard (**Table 4**). Similar results were reported by [44] after evaluating the inverse relationship between the phytotoxicity of trifluralin, atrazine, fluometuron, chloramben and propachlor with organic matter. Xu and Coventry [45] reported that the addition of organic matter to the soil contributed to soil acidification through the nitrification process.

Nitrate (r = 0.57, p-value <0.001) and chloride (r = 0.34, p-value <0.05) concentrations were positively correlated with depth in the AZ orchard (**Table 4**). The inverse relationship between chloride concentration with clay (r = -0.36, p-value <0.05) was likely the result of anion exclusion during transport through soil profile [46].

Alonso et al. [1] reported that the sorption of indaziflam increased with increasing organic matter content. Koskinen et al. [47] after evaluating the sorption and desorption of herbicides classified as weak acids in different soil types also reported that the sorption of herbicides is greater in soils with high organic matter content. Indaziflam and organic matter content showed a positive trend in this study, however no significant correlation was obtained. This is likely due to the low soil organic matter content and small sample size.

Previous studies have reported that soils with high clay content better preserve organic matter compared to soils having low clay content [48, 49]. The combined effect of clay and organic matter on the sorption of indaziflam could facilitate the degradation process if favorable abiotic and biotic conditions are present. Kah et al. [50] evaluated the degradation and sorption of six acidic and four basic pesticides in different soils and reported a positive correlation between the clay content and degradation rate for most of the pesticides.

Clay content and indaziflam showed a negative trend, however a non-significant correlation was obtained. A previous study reported that the sorption of indaziflam was negatively correlated with the pH and clay content of a mollisols [1]. The influence of net negatively charged clay minerals and pH on the CEC could be responsible for the repulsion of indaziflam from sorption sites. A study conducted by [5] showed an increase in CEC with increasing organic matter that at the same time reduced the toxicity of indaziflam to hybrid bermudagrass in sand cultures. The CEC of organic matter increases with soil pH, however, the sorption of indaziflam to organic matter could be due to the triazine amide group of indaziflam [5].

The present study evaluated the effect of soil properties on the occurrence and distribution of indaziflam under field conditions in two pecan orchards in semi-arid climate. Indaziflam was not detected in the majority of the soil samples and was on accord with the half-life. Despite limited analytical data from NM orchard, this study provided indications that indaziflam’s fate and transport behavior is not uniform in a field and can be influenced by the differences in soil properties. This work can serve as a baseline study for future investigations related to the fate and transport of indaziflam under field conditions. Further studies are required to evaluate dissipation mechanism of indaziflam in orchards and to determine whether dissipation is dominated by degradation, leaching or both. Additional studies are needed to evaluate the effect of variability of soil properties on the dissipation of indaziflam and breakdown products to control and minimize adverse impacts on crops in semi-arid areas.

## Conclusion

Indaziflam was detected in few soil samples collected from both pecan orchards approximately one year after the application of indaziflam. This was consistent with the half life of indaziflam and is good for maintaining the soil and environmental quality. The detection frequency of indaziflam was higher in AZ orchard than in NM orchard probably due to the differences in soil management practices and sand content. Indaziflam was mostly detected in areas where pecan trees were unimpacted. The lower organic matter content in impacted areas suggests that the leaching of indaziflam could have been greater as a result of lower sorption potential. No correlation was observed between silt content and depth in both orchards. However, clay and organic matter contents were negatively correlated with depth. No significant correlations were obtained between indaziflam and soil properties in AZ orchard. Further studies are required to evaluate the dissipation of indaziflam and breakdown products in different soil types.
